# Leveraging Technology and Theory to Change Health Behaviors, Close Gaps in Health-Related Social Needs, and Increase Enrollment in the National Diabetes Prevention Program

**DOI:** 10.5888/pcd22.240284

**Published:** 2025-03-13

**Authors:** Sara S. Johnson, Patricia H. Castle, Sasha Bosack

**Affiliations:** 1 ProChange Behavior Solutions, Narragansett, Rhode Island

## Abstract

**Purpose and Objectives:**

Although progress has been made in scaling up the National Diabetes Prevention Program Lifestyle Change Program (National DPP LCP), innovative engagement strategies are needed.

**Intervention Approach:**

This implementation evaluation leveraged and combined technology, behavior change theory, and community-based participatory design approaches to develop, deploy, and evaluate a 6-month, bilingual, tailored text message–delivered program (*bRIght communities*) to increase 1) readiness to engage in key behaviors for diabetes prevention, 2) engagement in services that address health-related social needs to reduce barriers to participation, and 3) readiness to enroll in the National DPP LCP.

**Evaluation Methods:**

We implemented a statewide, multichannel recruitment strategy from May through October 2022 and recruited 432 community members (62.3% White, 26.0% Hispanic, 6.2% Black) who received up to 6 months of tailored text messages. Six months postenrollment, 273 participants completed an online follow-up survey. Among those who did not complete the survey, responses from the last texting session were used for pre/post comparisons.

**Results:**

Matched pre/post analyses (using *t* tests and McNemar tests) indicated that *bRIght communities* had a significant impact on daily consumption of fruits and vegetables (d = 0.43); weekly physical activity minutes (d = 0.48); resilience (d = 0.26); food insecurity (*P* < .001); transportation concerns (*P* < .001); and perceptions of feeling unsafe exercising in one’s neighborhood (*P* < .001). Nearly 68% of participants with or at risk for prediabetes were in the precontemplation stage for enrolling in the National DPP. Overall, 30.3% of participants in *bRIght communities* moved forward at least 1 stage of change.

**Implications for Public Health:**

Interactive, theoretically driven tailored text messaging represents a promising approach to increasing awareness of prediabetes risk, readiness to enroll in the National DPP LCP, participant engagement, and health behavior change. Providing links to existing geographically matched community resources reduced health-related social needs that can present barriers to participating in the National DPP LCP. The results also provide insights to inform the design and development of other population-based tailored text-delivered interventions.

**Summary**

**What is already known on this topic?**

Innovative strategies are needed to increase engagement with the National Diabetes Prevention Program (National DPP) lifestyle change program (LCP).

**What is added by this report?**

We conducted a systematic evaluation of an interactive, tailored text-messaging program to address the awareness and engagement continuum for the National DPP (ie, identify risk for prediabetes, address health-related social needs that present barriers, tailor messages to increase readiness to participate, and facilitate referral).

**What are the implications for public health practice?**

Text messaging represents a promising approach to increasing readiness for and reducing barriers to patients’ engagement with the National DPP.

## Introduction

More than 1 in 3 US adults have prediabetes (1), and more than 80% of those who have prediabetes don’t know that they do (2). Having prediabetes increases the risk of developing diabetes and cardiovascular disease, as well as the risk of death due to cardiovascular disease (3)**.** One of the Healthy People 2030 objectives is to reduce the proportion of Americans who are unaware that they have prediabetes (4). To increase public awareness, the Centers for Disease Control and Prevention (CDC), in collaboration with other national organizations, launched the National Prediabetes Awareness Campaign (5) to make Americans aware of the risk factors and symptoms of prediabetes, connect people with the Diabetes Risk Screening Test, and link people who have prediabetes with the National Diabetes Prevention Program (National DPP).

The National DPP provides a structured, evidence-based lifestyle change program (LCP) designed to prevent or delay onset of type 2 diabetes (6). Although progress has been made scaling the National DPP LCP nationally, enrollment remains a challenge (7); only 3% of adults with prediabetes have engaged (3). Innovative methods and strategies are needed to enroll a higher proportion of at-risk people, which is particularly relevant for underserved populations who are at high risk for developing diabetes and its complications (8,9).

Frequently cited and real barriers to participating in the National DPP LCP include those related to social determinants of health (SDOH), including lack of knowledge of the program, cost to participate, and lack of time and transportation (10–12). Although addressing health-related social needs can reduce barriers to participation (eg, transportation barriers are mitigated by the increasing availability of online and distance learning National DPP LCP classes), additional critical barriers to enrollment remain. Those barriers include lack of readiness to engage in the key behaviors targeted by the National DPP LCP (ie, physical activity, healthy eating, and stress management) and differing levels of readiness to engage with the program.

Providing dynamically tailored, population-based behavior change messages appropriate for all adults via a widely used communication channel (13) has the potential to raise awareness of prediabetes and to advance readiness to engage in key health behaviors for diabetes prevention, as well as — among those who are eligible — increase readiness to enroll in the National DPP LCP. An estimated 97% of people in the US text daily; the open rate of texts is 98% and 90% of texts are read within 3 minutes of being delivered (14). Text messaging therefore represents a promising and powerful communication channel through which interventions can boost confidence and provide personalized reminders. The emergence of text-message interventions for health behavior change (15) has largely been uninformed by rigorous behavior change science. For their potential to be realized, behavior change theory must be at the foundation of these communications to tailor the behavior-change strategies based on recipients’ readiness to change and levels of self-efficacy. Deploying effective text-messaging campaigns requires a systematic approach to integrating best practices of behavior change science (eg, stages of change from the Transtheoretical Model [16]; social cognitive theory), as well as reliance on other robust communication frameworks (eg, principles of “pre-suasion” [17]). Pairing ongoing tailored behavior change micro-communications with screening for health-related social needs and localized referrals also has the potential to be particularly effective.

Although text messaging has been explored as an adjunct to the National DPP LCP (18,19) or as a delivery mechanism (20) for historically low-resourced communities, no systematic evaluations of efforts to use interactive text messaging to address the awareness and engagement continuum for the National DPP LCP appear to exist. To our knowledge, no studies have evaluated the effectiveness of a theoretically grounded short-message service (SMS)–delivered behavior change intervention that simultaneously enables identifying risk for prediabetes, addressing health-related social needs that present barriers to participating, tailored behavior-change reminders to increase readiness to participate, and geographically matched referrals to available recognized entities. This article addresses that gap by presenting the results of a 24-month initiative that involved 5 months of formative research, 5 months of intervention development, a 12-month demonstration project, and 2 months of data analyses.

## Purpose and Objectives

We leveraged technology, behavior change theories, and community-based participatory design approaches to develop, deploy, and conduct a statewide evaluation of a text message–delivered, bilingual, tailored behavior change program. This implementation evaluation examined whether this type of interactive, theory-driven technology solution could increase 1) readiness to engage in key behaviors for diabetes prevention, as indicated by forward stage progress from one stage of readiness to the next; 2) engagement in community services and resources that address health-related social needs to reduce barriers to participating in the National DPP LCP; 3) readiness and self-efficacy for enrolling in the National DPP LCP; and 4) enrollment in the National DPP LCP.

## Intervention Approach

In keeping with the Achieving Health Equity and Systems Transformation through the Meaningful Community Engagement Model (21), a community-based participatory design process was used to ensure maximum potential for engagement, successful implementation, and impact on health equity. Formative research was conducted from late 2021 through early 2022. Extensive formative input was obtained from 16 English- and Spanish-speaking participants from Rhode Island communities with high social vulnerability indices (SVI) through a series of three 1.5-hour interviews. Potential interview participants were invited via word-of-mouth referrals from community health workers and partners from community-based organizations serving communities with high SVI (eg, Providence, Pawtucket). Their feedback was combined with insights from 8 community health experts (eg, representatives of agencies from Rhode Island Health Equity Zones and federally qualified health centers, Master National DPP Trainers) who participated in five 2-hour meetings. In addition to the 8 community experts, 2 members from the Rhode Island Department of Health participated in key advisory meetings (ie, the interim Diabetes Prevention Program coordinator for the state and the Community Health Network manager). Community health experts were invited via word-of-mouth invitations following introductions through the statewide National DPP Stakeholder network or the state’s Health Equity Zones. Key participants and community members provided feedback on all aspects of program design, content development and promotional strategies, and resources available to help users with health-related social needs. One key contribution was the naming of the program. With the intention of having a program name that was tied to a superordinate goal with broad appeal, the name initially proposed to community members and community health experts was thRIve. Their input, that the program name should evoke a sense of connection and community to generate interest and still be localized, transformed the program name to *bRIght communities.* The updated name addressed participants’ concern that thRIve was too focused on individual versus community well-being and that bRIght had a Spanish cognate (*comunidades bRIllantes*) that maintained the local reference to Rhode Island.

The insights gleaned were combined with the feedback of 15 additional English- and Spanish-speaking community members who participated in a 10-day usability test of *bRIght communities* to inform the intervention development. Usability testers participated in a 30-minute video meeting, during which they were asked to enroll in the SMS program to ensure that they understood that enrollment required texting the keyword to the designated phone number. Usability testers then completed the first *bRIght communities* session. They were asked to think out loud about their user experience. Ten days later, a 1.5-hour follow-up meeting enabled usability testers to give feedback on the texts they received, the reminders to do their next assessment, the study landing page, a selection of recruitment posters, and their likelihood of recommending it to a friend. Usability testers were given a $50 gift card for their time. Revisions to the program based on usability testing feedback included adding images to the program (eg, animated gifs to illustrate what a cup of fruit and veggies looks like), clarifying questions and intervention messages, and encouraging participants to save the study telephone number as a contact.

Three of the main factors considered in intervention development were how to ensure that *bRIght communities* was engaging a broad sample of adults in a conversation about maximizing their health and well-being; could reduce barriers to improving health; and would increase awareness of prediabetes risk. Enrolling any adult created 1) an opportunity to deliver individually tailored feedback to increase readiness to engage in key diabetes prevention behaviors (ie, healthy eating and exercise), 2) a mechanism to identify and address unmet health-related social needs; and 3) an avenue of communication with people who may not be aware of having prediabetes.

**Figure 1:**
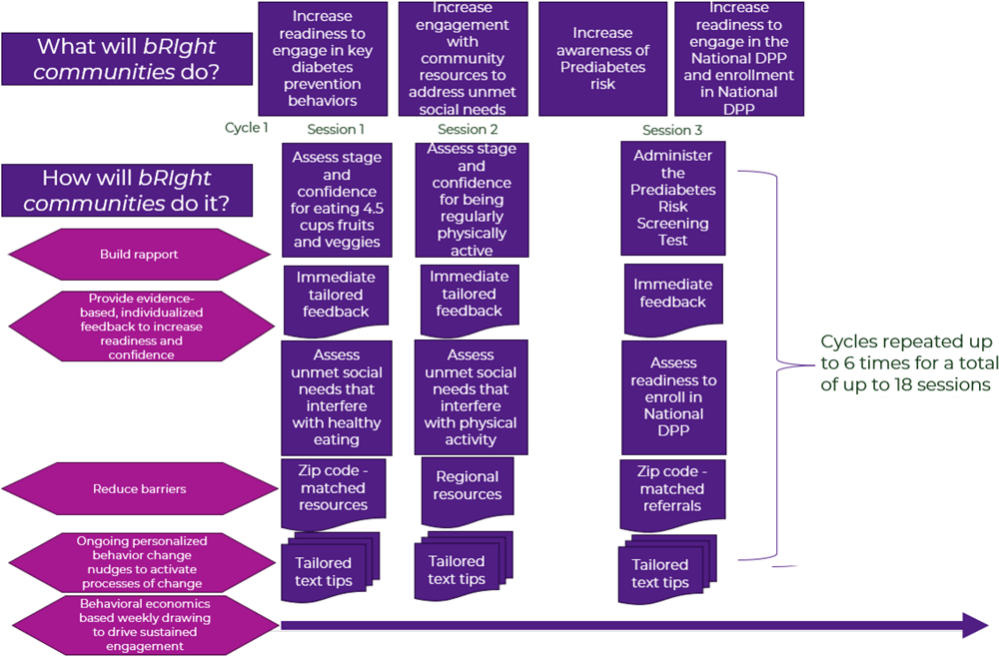
Range of objectives and strategies of *bRIght communities.*

Another equally crucial factor was ensuring that *bRIght communities* could personalize both the assessment and the intervention messaging to meet the needs of each person. The foundation for the tailoring technology driving the interactive text messaging was the Transtheoretical Model of behavior change (TTM) (22,23). The TTM is a model that frames readiness to change as a continuum of 5 stages of change: 1) Precontemplation = not yet ready; 2) Contemplation = getting ready; 3) Preparation = ready; 4) Action = recently adopted a new behavior; and 5) Maintenance = adopted a behavior more than 6 months ago and is feeling more confident about sustaining it (16,24). More than 35 years of research on the TTM have identified specific principles and processes (ie, strategies) of change that work best in each stage to facilitate progress (24–27). This evidence-based framework informs tailored feedback that is more likely to be remembered, considered personally relevant and credible, and to change behavior. Meta-analyses found that health interventions tailored to stage of change produced significantly greater effects than those not tailored to stage (24,25). Thus, the TTM was used as the key unifying theoretical framework for *bRIght communities* (Figure 1).

*bRIght communities* is a customized and individually tailored user journey in a fully automated SMS-delivered experience based on each participant’s readiness to engage in various health behaviors, level of self-efficacy for each, health-related social needs, risk for prediabetes, and readiness to enroll in the National DPP LCP. Users were invited to text a keyword (ie, “bright”) to a local number to complete a brief screening to confirm eligibility. Through a series of interactive text messaging sessions, preprogrammed decision rules operated to dynamically present the appropriate questions and personalized feedback for each user based on their responses. The immediate tailored feedback users were given was supplemented by customized text messages. Intervention delivery from an automated text messaging delivery platform was standardized, ensuring high treatment fidelity, as well as being cost-effective and easily accessible among anyone regardless of their device type (eg, flip phone).

*bRIght communities* was split into 10-day sessions that included assessment questions related to the focus of that session. During the initial onboarding session, which lasted approximately 5 to 7 minutes, users were asked to report their zip code and their current daily consumption of fruit and vegetables. Based on responses, users were then assessed on their stage of readiness and self-efficacy for consuming 4.5 cups of fruits and vegetables daily (the recommendation from the Dietary Guidelines for Americans). Users then received tailored feedback. Users in Precontemplation, Contemplation, or Preparation were asked about social needs related to fruit and vegetable intake. Users who confirmed food insecurity or transportation barriers were provided with immediate zip code–matched community resources to help close gaps. Users’ responses were used to queue 30 days of tailored text message tips that were matched to their level of readiness to consume sufficient fruits and vegetables and to their specific constellation of health-related social needs (if any). The schedule for delivery of those texts was also variable and dependent on the user’s stage of change.

To maintain engagement, participants completing Session 1 became eligible for a “regret style” contest for the duration of the study. The weekly drawing used multiple principles of behavioral economics (eg, loss aversion, tendency to overestimate small probabilities). Every 10 days, anyone who had responded to at least 1 text was eligible to win one of ten $10 gift cards or one $100 gift card based on a random drawing. In past studies, adding a “regret contest” increased enrollment, adherence, and long-term engagement (28–31).

During Session 2, users were asked to report their weekly minutes of physical activity. Users were then asked about their readiness and confidence to engage in regular physical activity (ie, 150 minutes of moderate physical activity per week). They received immediate tailored feedback. Any user who confirmed concerns about perceived neighborhood safety or that childcare presented a barrier to being physically active were given feedback and regional resources to address those concerns. Up to 30 days of tailored text messages were then queued to be sent based on a cadence that was determined by their stage of change.

During Session 3, the Prediabetes Risk Test (32) was administered. People with or at risk for prediabetes were provided with information about the National DPP LCP and asked about their past or current participation. People who had never participated or who had previously dropped out were then asked about their readiness and confidence to participate in the National DPP LCP. Immediate feedback based on stage of change and confidence was presented in conjunction with a zip code–matched referral to the geographically closest National DPP LCP and the state Community Health Network. Tailored messages based on readiness to participate were then queued up for 30 days and delivered based on stage.

The logic and decision rules allowed the cycles of 3 sessions to recur every 30 days. Stage of change for each respective behavior was reassessed in each session to allow feedback to be dynamically retailored over time. People with persistent health-related social needs received new resources at subsequent sessions.

A 12-month statewide demonstration project was conducted in Rhode Island to evaluate the feasibility, acceptability, and effectiveness of *bRIght communities.* Recruitment began in May 2022 and continued on a rolling basis for 6 months. Multichannel, grassroots promotional strategies (ie, radio, online, email, flyers, and in-person) were used to recruit participants. These methods included the dissemination of promotional materials statewide via community partnerships, direct community outreach, and community events. Rhode Island residents were eligible to participate if they were aged at least 18 years, were not pregnant, and had not been diagnosed with diabetes. Users who were screened out based on 1 or more of the exclusion criteria were provided with local resources. Although recruitment efforts and materials were disseminated in every town and city in the state, they were most heavily focused in cities with the highest social vulnerability. Identifying a variety of nontraditional community channels such as churches and car washes allowed *bRIght communities* to reach traditionally underserved and hard-to-reach populations. Various community partnerships were developed and cultivated to facilitate and strengthen the association of *bRIght communities* with existing community-based organizations with whom residents already had a trusted relationship.

## Evaluation Methods

Self-efficacy (ie, confidence) to enroll in the National DPP LCP was the primary outcome measure used to evaluate the effectiveness of *bRIght communities* texting program, as it is among the measures recommended in the Centers for Disease Control and Prevention’s (CDC’s) health communication and marketing toolkit (33). Participants were asked to respond to the question “How confident are you that you will take part in a DPP?” on a 5-point Likert scale ranging from 1 being “not at all confident” to 5 being “extremely confident.” Based on an initial power calculation assuming a small effect (d = 0.3) on confidence and a 75% retention rate, the minimum recruitment goal was 416 participants. Secondary outcomes included readiness to engage in the health behaviors addressed in the National DPP LCP (ie, healthy eating and physical activity) and rates of health-related social needs. Increasing readiness to engage in these health behaviors is crucial to promote overall health and well-being and to prevent chronic illnesses among people not at risk for prediabetes. Furthermore, increasing readiness for these behavior changes and addressing health-related social needs could play an important role in increasing sustained engagement and success in the National DPP LCP.

Participants interacted in their preferred language and received up to 6 months of tailored text messages. On day 180, each user (regardless of number of sessions completed during the intervention) received an SMS message to complete the final follow-up survey and a link to a secure online platform. Participants received a $25 grocery store or Amazon gift card for completing the onboarding assessment and another $25 gift card for completing the final assessment 6 months later. The research protocol was reviewed and approved by the Pro-Change Behavior Systems Institutional Review Board, which has federal-wide assurance. All data were stored on secure servers that were regularly audited to ensure compliance with rigorous data privacy standards.

A robust evaluation was conducted in the context of a single group, pre/post comparison over a 6-month period. Paired *t* test and McNemar tests were used to compare baseline data (ie, responses at onboarding) to the last available data for each participant. The McNemar test is a nonparametric test used to analyze paired nominal data (34). For some participants (n = 273), the “last” available data was the 24-week final follow-up evaluation. For others, the “last” available observation was a response within a session of *bRIght communities.* Consideration was given to reporting the pre-to-post comparison of onboarding to final follow-up assessment data, but sample sizes were larger when all available data were used, and results were remarkably similar. Thus, to maximize power, all available data were used. The last value carried forward was used for any participants who had postbaseline data but no final follow-up assessment data. Analyses were conducted by using SPSS (SPSS Inc). When reporting paired *t* tests, estimates of effect size are presented in Cohen’s d, with d = 0.2 representing a small effect, d = 0.5 representing a medium effect, and d = 0.8 representing a large effect (35). Although this study was not powered to do subgroup analyses by race or ethnicity, the proportion of White participants who were enrolled in the National DPP LCP at onboarding versus the last session was compared with that of non-White participants.

## Results

**Figure 2:**
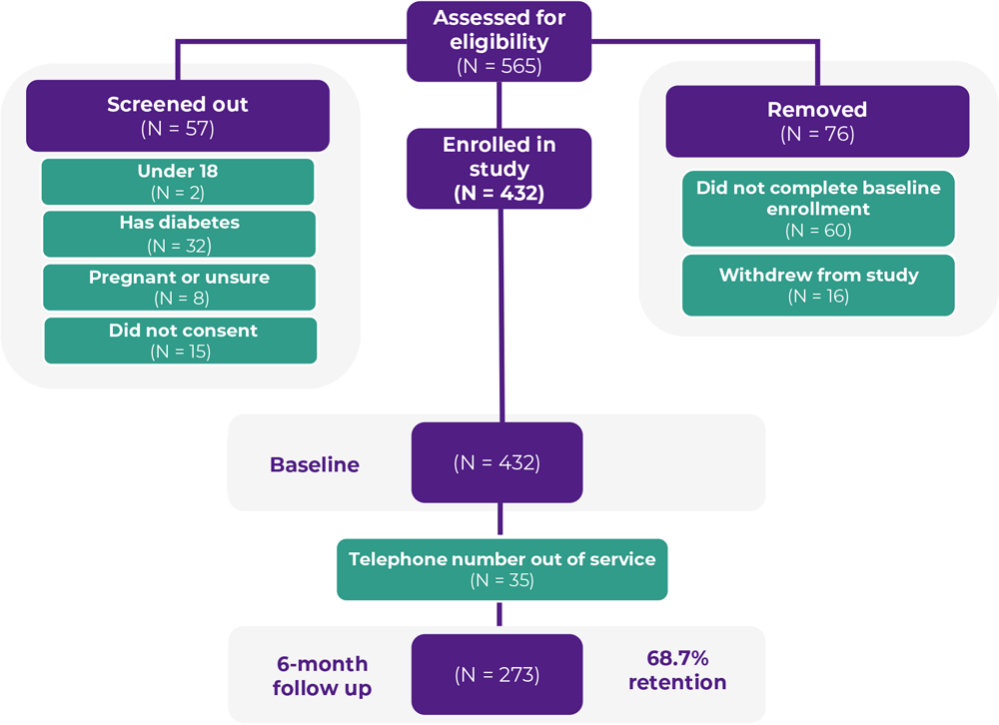
Eligibility and participation of *bRIght communities* participants.

A total of 432 participants enrolled in the study. During the study period, 35 participants had a mobile number that went out of service. Thus, 397 participants were invited to complete the 6-month follow-up assessment. A total of 273 did so, representing a 68.7% retention rate (Figure 2).

The average age of participants was 40.1 years (SD, 13.4 y), 78.2% were female, and 70.3% had a body mass index in the overweight or obese range. Race and ethnicity were assessed only at the follow-up assessment. Among the 273 respondents, 62.3% were White, 26.0% were Hispanic, 6.2% were Black, and 5.5% reported another race. 

Users could complete as many as 18 sessions over 6 cycles (ie, 6 months). A total of 119 users (27.5%) initiated the sixth cycle (ie, Session 16). On average, users completed 8.6 sessions of *bRIght communities.*

### Primary outcome: readiness and self-efficacy to enroll in the National DPP LCP

A total of 300 participants completed the Prediabetes Risk Screening Test at the outset of Session 3 in Cycle 1. Approximately 31% (n = 93) had or were identified as being at risk for prediabetes based on the Prediabetes Risk Test. *bRIght communities* identified 58% of those at risk for prediabetes (n = 54) who were not aware they were at risk. The stage of change distribution for enrolling in the National DPP among individuals who had or were at high risk for prediabetes is depicted in Table 1.

**Table 1:** Readiness of People with Prediabetes (N = 93) to Participate in the National Diabetes Prevention Program (DPP) Lifestyle Change Program

Stage of change for enrolling in the National DPP	% of Participants
Precontemplation	67.9
Dropped out of a previous national DPP	3.6
Contemplation	8.3
Preparation	10.7
Waiting list for the National DPP	2.4
Action	3.6
Maintenance	3.6

The increase in confidence to enroll in the National DPP LCP from first to last session was not statistically significant (*t* = 1.33, *P = .*19, d = 0.15). Among people who were not extremely confident at the onboarding session and therefore had an opportunity to increase their confidence (ie, answered 1–4 on a 5-point scale), 29.5% had higher self-efficacy at their last session, with scores increasing from 1.9 to 2.1 points.

### Enrollment in the National DPP LCP

Significantly more participants had enrolled or asked to enroll at the last session than at the first session (*P = .*01). At onboarding (first session), 7.2% of participants reported already being enrolled in a National DPP LCP, and 2.4% reported being on a waiting list for a National DPP LCP. At the last session, those numbers had increased: 15.4% reported being enrolled in a National DPP LCP, and 4.8% reported being on a waiting list for a National DPP LCP.

Among White participants, 5.3% were enrolled in the National DPP LCP at onboarding and 15.8% reported being enrolled or on a waiting list at the final time point. Among non-White participants, 21.1% were enrolled in the National DPP LCP at onboarding and 36.8% were enrolled or on a waiting list at the last time point.

### Secondary outcomes: readiness to engage in health behaviors for diabetes prevention

#### Fruit and vegetable consumption

More than 92% of participants were not consuming 4.5 cups of fruit and vegetables each day at their first (onboarding) session. Among them, 7.5% were in Precontemplation, 2.5% were in Contemplation, and 82.1% were in Preparation. The proportion of participants in Action and Maintenance (consuming adequate fruits and vegetables) at onboarding was compared with the proportion in Action and Maintenance at the last session. Significantly more participants were in Action and Maintenance at their last session (23.8%) compared with at onboarding (7.8%, *P* < .001).

Daily cups of fruits and vegetables consumed increased significantly from first to last session. At onboarding, participants consumed an average of 2.43 cups per day (SD, 1.63). At the last session, the average had increased to 3.21 (SD, 1.70; *t* = 7.76; *P* < .001; d = 0.43). Overall, 60% of participants had some increase in their daily consumption of fruit and vegetables.

Similar findings were seen among people with or at risk for prediabetes. From first session to last session, average daily cups of fruit and vegetables increased significantly from 2.17 cups to 3.00 cups (*t* = 4.42, *P* < .001, d = 0.47) among people with or at risk for prediabetes. Among those not at risk, average daily cups increased from 2.58 to 3.44 (*t* = 6.69, *P* < .001, d = 0.48). The increase in daily cups of fruit and vegetables was higher (mean increase = 0.86 cups, *t* = 5.42, *P* < .001, d = 0.53) for non-White participants than for White participants (mean increase = 0.73 cups, *t* = 5.05, *P* < .001, d = 0.39).

#### Physical Activity

At onboarding, 50% of the participants were engaging in at least 150 minutes of physical activity each week (ie, they were in Action and Maintenance). There was a small, nonsignificant increase (to 53.5%) in the proportion in Action and Maintenance at the last session (*P = .*30). Substantial improvements were noted among people who were not regularly physically active at the first session (ie, the participants in Precontemplation, Contemplation, or Preparation). More than one-third (34.3%) of them moved forward at least 1 stage of change.

A small, nonsignificant increase was found in self-efficacy for physical activity among people in the pre-Action stage from first (mean 3.20 [SD, 0.88]) to last session (mean = 3.37 [SD, 1.01], d = 0.15). A substantial increase was found in weekly minutes of physical activity among this group, with 59.4% of participants reporting an increase. Weekly minutes increased from 58.31 to 113.34 from first to last session (*t* = 5.77, *P* < .001, d = 0.48).

Among people who were at risk for or who had prediabetes and were in a pre-Action stage at onboarding, weekly minutes of physical activity increased significantly from an average of 46.88 to 89.02 (*t* = 3.48, *P* < .001, d = 0.52). The increase in weekly minutes of physical activity was higher (mean increase = 74.30 min, *t* = 4.29, *P* < .001, d = 0.67) for non-White participants than for White participants (mean increase = 43.03 min, *t* = 3.73, *P* < .001, d = 0.40). Overall, 56% of White participants increased their weekly minutes of physical activity, and 66% of non-White participants did so.

### Secondary outcomes: health-related social needs

**Figure 3:**
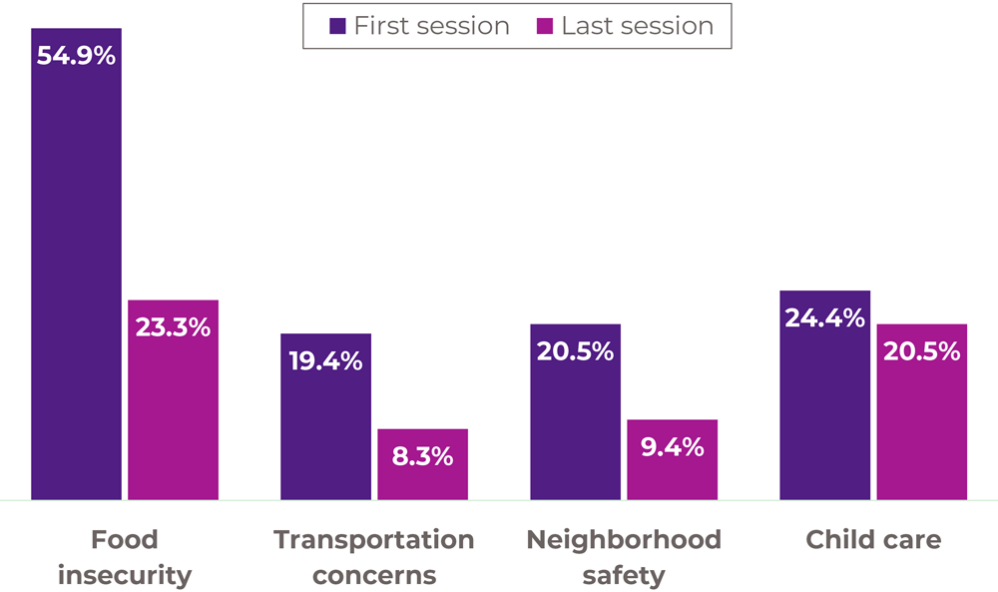
Comparison of proportion of *bRIght communities* participants reporting health-related social needs from first to last session.

McNemar tests showed significant reductions in food insecurity (*P* < .001), transportation concerns that make it difficult to obtain healthy food (*P* < .001), and perceptions of feeling unsafe exercising in one’s neighborhood (*P* < .001) from first to last session among participants. A reduction in the proportion of participants endorsing that problems with childcare make it difficult to exercise (*P = .*06) was seen from first to last session (Figure 3).

People with prediabetes had similar meaningful reductions in health-related social needs to those who did not report having or being at risk for prediabetes (Table 2). The proportion of those with health-related social needs was also compared between participants who reported being White versus participants who reported any other race or ethnicity. Gaps in health-related social needs were closed for all participants, but non-White participants were more likely to endorse health-related social needs (Table 2).

**Table 2:** Comparison of Proportion of *bRIght communities* Participants Reporting Health-Related Social Needs, From First to Last Session, by Prediabetes Status and Race and Ethnicity

Unmet social need	Participants with prediabetes, %		Participants without prediabetes, %		White participants, %		Non-White participants, %	
	Onboarding	Final session	Onboarding	Final session	Onboarding	Final session	Onboarding	Final session
**Food insecurity**	50.0	27.5	57.2	21.4	36.7	12.2	77.8	30.6
**Transportation**	15.0	7.5	21.4	8.7	10.1	5.0	30.6	8.3
**Neighborhood safety**	24.4	13.3	18.3	7.3	16.3	7.5	30.3	12.1
**Childcare**	17.8	13.3	28.0	24.4	18.8	15.0	42.4	36.4

### Acceptability data

Participants rated *bRIght communities* with an average star rating of 4.2 (of 5), with 79.2% giving 4 or 5 stars. More than 89% reported that they read most or all of the texts, and 76.6% reported that the program gave them “new things to think about.” Nearly 62% reported that the texts they received were personalized for them, and 71.8% reported that the program could help them be healthier.

In response to what participants liked best about *bRIght communities*, common themes were that it provided helpful, informative tips and reminders. The texts reminded users to engage with the program but also acted as a gentle, continual reminder of the importance of their daily health behaviors (eg, one participant said, “Gentle reminders to eat healthy!”). Another common theme was the interactive tailoring (eg, “personalized information and positive feedback”).

## Implications for Public Health

The data confirm that using theoretically driven, interactive, tailored text messaging can increase awareness of prediabetes risk, readiness to enroll in the National DPP LCP, and engagement. Overall, 30.3% of participants in *bRIght communities* moved forward at least 1 stage of change for enrolling in the National DPP LCP.

The stage of change distribution for readiness to enroll in the National DPP LCP among people who are eligible (ie, 90.5% in a pre-Action stage and nearly 70% of participants in the Precontemplation stage) underscores how critical it is to provide tailored health behavior change communications about the National DPP LCP to increase readiness. Action-oriented messages are not well-matched to individuals in the Precontemplation stage of change. The goal for people in Precontemplation is to promote forward stage progress to Contemplation (16). *bRIght communities* had an effect on progression out of the stable Precontemplation stage: nearly 25% of those in Precontemplation at the onboarding session moved forward at least 1 stage of change or took action to enroll in the National DPP LCP. Forward stage movement is a positive intermediate success metric; moving forward at least 1 stage can as much as double the probability that a person will move to the Action stage within the following 6 months (36).

The results also affirm and are consistent with past studies that speak to the ability of health communications based on the TTM to create behavior change on key diabetes prevention behaviors (23,27). Here, however, the delivery channel was brief, interactive texting, a novel intervention channel for a tailored, behavior-change intervention for multiple behaviors. The proportion of participants who increased their daily consumption of fruit and vegetables and the medium effect size of the increase from 2.4 to 3.2 compare favorably to a meta-analysis of 19 studies on e-health interventions to improve fruit and vegetable consumption in which the overall effect size was small (d = 0.26) (37). Similarly, the medium effect on weekly minutes of physical activity (with an increase of 55 minutes per week) compares favorably to previous research on physical activity interventions. One systematic review and meta-analysis (n = 16,476) reported an average increase of 14.2 minutes for interactive online physical activity interventions (38), and another systematic review and meta-analysis of 46 randomized trials including more than 16,000 participants reported that physical activity interventions delivered by health care providers in primary care resulted in an increase of 14 to 24 minutes of moderate physical activity a week (39).

One of the key objectives of *bRIght communities* was to close gaps in health-related social needs by providing localized referrals to community health services. Providing links to existing geographically matched community resources reduced critical health-related social needs that can present barriers to participating in the National DPP LCP. Our results emphasize the importance of navigation and support services (eg, community health workers) in communities experiencing more health-related social needs.

Intervention dose is an important consideration worth mentioning. Program users can benefit from variable user experiences. Although users could engage in up to 18 sessions over the 6 months, some elected to participate in fewer sessions of *bRIght communities.* The users who engaged in fewer sessions, however, need not be considered “dropouts,” in that some achieved the same outcomes as those who engaged in more sessions. One user, for example, interacted for 5 sessions and progressed from Precontemplation to Preparation for taking part in the National DPP — the same outcome achieved by another user who engaged in 16 sessions. Future research should explore dose response in more depth.

The strengths of this approach include the heavy reliance on participatory design and robust theoretical frameworks, as well as the number and robustness of partnerships with an array of community-based organizations and boots-on-the-ground recruitment efforts. This approach is repeatable, scalable, and generalizable to other communities and can inform the design and development of other population-based tailored SMS-delivered interventions. The automation of the decision rules and SMS delivery ensures that disseminating the tailored behavior change feedback to larger communities is easily achieved. The effort required to adapt *bRIght communities* to different contexts or populations is identifying and matching the recognized entities offering the National DPP and the resources for health-related social needs to the region and additional language translations as needed. Future research could also explore additional strategies for maintaining engagement to supplement the regret contest.

These results also highlight opportunities for improving the design of SMS programs, such as adding even more refined tailoring; more images; more links to resources; links to recipes and cooking demonstrations; reminders to users at onboarding and on the landing page of the importance of reconnecting with us if their number changes or they get a new mobile number; and a more spaced series of reminders at a less frequent interval to re-engage users. Another potential enhancement that warrants further exploration would be to enable users to set the desired frequency for text messaging from the outset or to allow the selection of a less-frequent cadence among users who have not yet returned for a follow-up session as a mechanism for re-engaging.

Limitations of this implementation evaluation include the self-selection bias operating on who initially elected to enroll and who completed a follow-up assessment. The wide-ranging recruitment efforts and extensive efforts to capture follow-up mitigate these concerns to a certain degree. The reliance on a single-group design is also a limitation in that we could not eliminate questions about the role of confounding factors that could have influenced the outcomes. Future studies should conduct randomized trials to address questions about the potential influence of secular trends.

Given the cost effectiveness of text messaging and high acceptability ratings given to *bRIght communities,* interactive tailored texting rooted in behavior change science represents a promising approach to improving population health and increasing enrollment in the National DPP LCP. This approach offers the opportunity to increase awareness of risk, as well as awareness of and, ultimately enrollment in, the National DPP LCP, particularly among underserved people living with prediabetes.
